# A case report of Hyper‐IgD syndrome in a 5‐year‐old girl with recurrent fever, skin rash, and arthralgia; novel MVK mutation (*C.298G>A*)

**DOI:** 10.1002/ccr3.8833

**Published:** 2024-04-30

**Authors:** Pooneh Tabibi, Reza Shiari, Shabnam Hajiani Ghotb Abadi

**Affiliations:** ^1^ Department of Pediatric Rheumatology, Mofid Children's Hospital Shahid Beheshti University of Medical Sciences Tehran Iran; ^2^ Faculty of Medicine Shiraz University of Medical Sciences Shiraz Iran

**Keywords:** hyper‐IgD syndrome, mevalonate kinase, periodic fever, skin rash

## Abstract

**Key Clinical Message:**

This case highlights the potential for later‐onset Hyper‐IgD syndrome (HIDS) even beyond infancy. Clinicians evaluating children with recurrent fever, skin rash, and arthralgia should consider HIDS in the differential diagnosis, regardless of age. Early suspicion and genetic testing can lead to a timely diagnosis and targeted therapy with Anakinra, significantly improving patient outcomes.

**Abstract:**

Hyper‐IgD syndrome (HIDS) is a rare autosomal recessive autoinflammatory disorder characterized by recurrent episodes of fever, lymphadenopathy, arthralgia, diarrhea, abdominal pain, and skin rash. In this case report, we present a 5‐year‐old girl from Tajikistan with a mutation in the mevalonate kinase (MVK) gene, which is consistent with a diagnosis of HIDS. The clinical symptoms of the patient are described, along with immunological, hematological, and biochemical findings collected from the evaluation in the rheumatology clinic. Additionally, whole‐exome sequencing revealed a heterozygous missense variation in exon 4 of the MVK gene. The diagnosis of HIDS in this case occurred at a later age than typically observed, emphasizing the importance of considering this condition even in older patients. This report highlights the broad clinical phenotype of MVK and the need for increased awareness among healthcare professionals regarding its clinical presentation and management.

## 1 INTRODUCTION

Hyper IgD syndrome (HIDS) is a rare autosomal recessive autoinflammatory condition, determined by recurrent febrile attacks associated with lymphadenopathy, arthralgia, diarrhea, abdominal pain, and skin rash.^1^, ^2^ This disorder was first described in 1984 by Jos van der Meer.^3^

This disease has an early onset, generally in infancy, and febrile crises recur at varying intervals. Fever flares have a sudden onset and last approximately 4–6 days. An attack begins with chills, and patients often complain of weakness, headache, nausea, and diarrhea.^1^, ^4^ The hallmark characteristic of the syndrome is the presence of recurrent episodes of fever and chills, often accompanied by skin rashes.^5^ HIDS is caused by a mutation in the mevalonate kinase (MVK) gene, which results in a lack of mevalonate kinase enzyme activity.^6^, ^7^ Mevalonate kinase is a key enzyme in isoprenoid biosynthesis and is involved in a wide range of essential cellular processes and the synthesis of cholesterol.^8^

According to our current knowledge of the genetics and pathophysiology of HIDS, the diagnosis can be made in a patient with frequent episodes of fever and the usual findings associated with recording mutations in MVK or high levels of mevalonic acid, a substrate of mevalonate kinase, in the urine of patients during attacks.^9^ Whereas before the discovery of the MVK gene as the cause of HIDS, the presence of high serum IgD levels was necessary for the diagnosis of HIDS.^10^ Today, the importance of increased IgD without genetic or biochemical findings remains uncertain.^9^

Early diagnosis and treatment of HIDS patients is momentous, because it prevents irreversible organ damage and improves the quality of life of patients.^11^ Management of HIDS focuses primarily on symptomatic relief of febrile episodes and prevention of complications. Nonsteroidal anti‐inflammatory drugs (NSAIDs) are considered as first‐line treatment for pain and fever associated with HIDS episodes. In some patients, immunomodulatory therapy may be recommended to help control the frequency and severity of episodes.^12^, ^13^

In this case report, we presented a girl with a long history of fever episodes and skin manifestations, in whom a mutation in the MVK gene could be identified.

## 2 CASE REPORT

### 2.1 Case history and examination

A 5‐year‐old girl, born of a non‐consanguineous marriage, presented with clinical indications of low to moderate‐grade fever persisting for the past 2 years, along with recurrent skin lesions and infections. The patient, a first‐born child of non‐consanguineous parents (G1P1L1), had a birth weight of 3800 g and current weight of 17 kg. Her date of birth was January 2017, and she had a history of normal neurodevelopment. She was a native of Tajikistan with no known history of specific diseases. There were no positive points in the family history regarding similar conditions.

Approximately 2 years ago, the patient started experiencing episodes of low to moderate‐grade fever and recurrent generalized skin lesions, affecting the trunk, abdomen, and face. The skin lesions exhibit a recurring pattern and resemble petechial rashes observed in certain areas of the body. She also complained of arthralgia. Initially, multiple physicians in Tajikistan suspected a dermatological condition.

However, due to the lack of improvement in the patient's symptoms, she was referred to BLK‐MAX Hospital in central Delhi in January 2023, with a history of fever persisting for a year and the recent development of pustular skin lesions over the past month. At BLK‐MAX Hospital, primary immunodeficiency was suspected, and further tests were requested. In August 2023, the patient was referred to the rheumatology clinic of Mofid Children's Hospital in Tehran, Iran.

During the evaluation in the rheumatology clinic, the patient's height and weight were measured at 100 cm and 17 kg, respectively. Physical examination revealed pallor, moderate‐grade fever with a sporadic pattern, and no skin lesions at the time of admission. There were no oral lesions, no signs of icterus, lymphadenopathy, organomegaly, and the chest examination was clear. The patient had a history of arthritis, arthralgia, and sore throat in the course of the disease. Immunology consultation was conducted, and an immunological examination, including complete blood count (CBC), CD markers, and immunoglobulin levels, was performed. The hematological, biochemical and immunological findings of the patient are provided in Table 1.

**TABLE 1:** Hematological, biochemical and immunological findings of the patient.

Variable	Value	Normal range	Variable	Normal range	Value
WBC (cell/mm^3^)	**18,300**	4000–12,000	Anti‐CCP	Negative	Negative < 10
Neutrophil (cell/mm^3^)	13,500	1500–8500	HLA‐B51	Negative	
Lymphocyte (cell/mm^3^)	3800	3000–9500	LDH	803	<746
Hb (gr/dL)	**7**	11.5–13.5	CPK	36	24–195
MCV (fl)	69.4	77–95	RETIC	1.5%	0.2–2
PLT (×10^3^/L)	650,000	150,000–450,000	Uric acid (mg/dL)	3.5	3–7
ESR (mm/h)	**118**	NL < 20	CRP (mg/L)	**57**	NL <6
IgM (mg/dL)	104	24–210	IgG (mg/dL)	1270	504–1464
IgA	272	27–195	IgE (IU/mL)	351	Up to 52
ANA	Negative	Negative<1.40			
RF	Negative	Negative<10	Stool calprotectin	145	NL < 50
Ferritin (ng/mL)	**861.3**	1.5–205	CH50 (mg/dL)	64	51–150
C3 (mg/dL)	236	90–180	C4 (mg/dL)	41	10–40
D‐dimer (g/mL)	3.1	Negative<0.5	Ant‐ds DNA (IU/mL)	<10	Negative <100
Fibrinogen (mg/dL)	797	200–400	ANCA/IFA	<1/20	Negative titer <1/20
CMV/IgM	05	Nonreactive<0.9	CMV/IgG	22.6	Reactive >1.1
Anti‐Beta‐2 glycoprotein‐IgG (au/mL)	2.3	Negative<10	Anti‐beta‐2 glycoprotein‐IgM (au/mL)	0.1	Negative <10
Lupus anticoagulant	71.9	33–41	HBs Ag	Nonreactive	
HIV	Nonreactive		Serum IgD	**45**	0.3–11
KFT	Normal		Excretion of mevalonic acid	**1.5**	0.1–0.7

*Note:* Bold indicate our patient laboratory data showed Leukocytosis, thrombocytosis, anemia, elevated acute phase reactants (ESR & CRP), high serum level of IgD and high mevalonic acid urine excretion.

Abbreviations: ANA, anti‐nuclear antibody; anti CCP, anti cyclic citrullinated peptide; Anti Ds DNA, anti double stranded DNA; CRP, C‐reactive protein; ESR, erythrocyte sedimentation rate; Hb, hemoglobin; HLAB27, human leukocyte antigen B5; Ig, immunoglobulin; KFT, Kidney Function Test; LFT, Liver Function Test; PLT, platelet; RF, rheumatoid factor; WBC, white blood cell.

**FIGURE 1:**
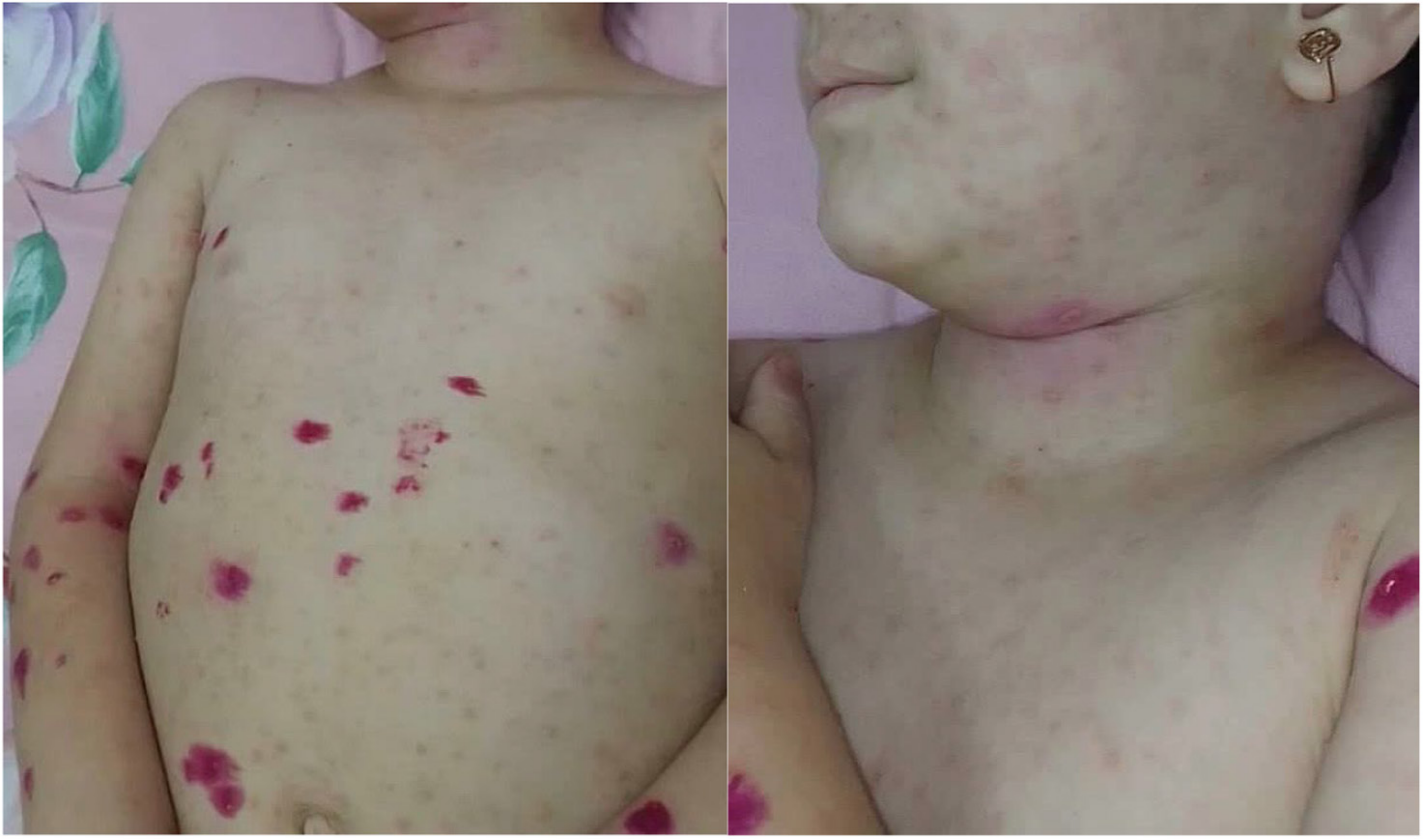
The skin manifestations in our patient.

Whole‐exome sequencing (WES) was performed on a blood sample from the patient. Variant interpretation of specific variants of interest was conducted following the guidelines of the American College of Medical Genetics and Genomics (ACMG). A heterozygous missense variation was identified in exon 4 of the MVK gene, resulting in the amino acid substitution of asparagine for aspartic acid at codon 100 (*𝘊*.*298𝘎>𝘈*; *𝘗*. *𝘈𝘴𝘱100𝘈𝘴𝘯*). This variant is classified as having uncertain significance according to the ACMG guidelines and is associated with the OMIM phenotype of Hyper IgD Syndrome, which is typically caused by homozygous or compound heterozygous mutations in the MVK gene (Table 2). The clinical symptoms of the patient are consistent with this phenotype. The skin manifestation (petechial like rash) of the patient is showed in Figure 1.

**TABLE 2:** The result of whole exome sequencing of the patient.

Gene	Location	Variant	Zygosity	Disease (OMIM)	Inheritance	Classification
MVK (+) (ENST00000228510.8)	Exon 4	C.298G>A (P.Asp100Asn)	Heterozygous	Hyper‐IgD syndrome (OMIM#260920)	Autosomal recessive	Uncertain significance

In our case, the patient presented with significantly elevated serum IgD levels (45 mg/dL) and mevalonic acid excretion in urine (1.5 μmol/mol creatinine), exceeding the age‐dependent reference range (0.1–0.7 μmol/mol creatinine for patients >1 year).

### 2.2 Treatment, outcome, and follow‐up

Based on our patient's clinical presentation, genetic confirmation of a heterozygous missense variant (*𝘊.298𝘎>𝘈*) in the MVK gene, and significantly elevated mevalonic acid levels in urine, a diagnosis of Hyper‐IgD syndrome (HIDS) was established. Due to the severity of her symptoms, including recurrent fever, skin lesions, and arthralgia, initial treatment included a short course of methylprednisolone pulse therapy (IV) at a dosage of 30 mg/kg/day for three doses, followed oral prednisolone at a dosage of 2 mg/kg/day, which was gradually tapered. We should have added one Biologic Disease‐modifying Medications to the treatment, due to the potential side effects associated with long‐term corticosteroid use. While canakinumab may be a viable treatment option for HIDS, it is currently not available in our facility. Therefore, Anakinra, an interleukin‐1 receptor antagonist, which was readily available was initiated. Notably, this resulted in a remarkable improvement in both our patient's symptoms and laboratory investigations. Our patient experienced a significant reduction in fever episodes. Skin lesions resolved completely, and the patient reported a marked improvement in joint pain and overall well‐being.

It is important to note that early diagnosis and treatment with interleukin‐1 inhibitors (Anakinra) may have contributed to the absence of amyloidosis complications in this case. However, long‐term monitoring is essential to assess for potential future complications.

## 3 DISCUSSION

HIDS is an autoinflammatory disorder determined by recurrent and periodic fever episodes occurring since early childhood. Fever episodes generally related to wide range of symptoms such as joint pain, abdominal pain, headache, enlargement of lymph nodes, diarrhea, and skin involvement.^13^, ^14^ In the current study, we reported the clinical manifestations of a girl with a mutation in the MVK gene, which is consistent with a diagnosis of HIDS. Patients with MVK gene reveal first fever episodes in early childhood (under 1 year old).^15^ In our patient, the onset of fever occurred at the age of 3 years old, which is rather late. The presentation of HIDS in this case occurred at a later age than typically observed, emphasizing the importance of considering this condition even in older patients. The nonspecific nature of the symptoms and the overlap with other conditions pose a diagnostic challenge, underscoring the need for increased awareness among healthcare professionals. Similar to our report, Yoshimura et al. reported a boy having periodic fever since 3 years and 2 months.^16^

In the study of Tsitsami et al., the recurrent and periodic nature of the disease in a 7‐year period indicated periodic fever syndrome. A number of periodic fever syndromes were excluded based on a combination of laboratory findings, genetic testing, and clinical features. Serum IgD values were more than 11 mg/dL on more than two occasions with an interval of 1 month. Although this does not fully confirm the diagnosis of HIDS, the patient's fever patterns and clinical symptoms suggested the HIDS for the patient. Contrary to our patient, and based on a complete analysis of the MVK gene, no mutation was found.^17^

The skin lesions of HIDS are generally erythematous maculopapular rashes, urticarial, and aphthous ulcers mimicking Behçet's disease.^5^

It is significant to differentiate the skin rash associated with HIDS from other skin diseases that show similar manifestations of fever and rash. The initial diagnosis for our study patient was a skin disease, and the patient was treated accordingly. In study by Omoyinmi et al. a 2‐year‐old boy who had suffered from frequent episodes of fever since early infancy with maculopapular/petechial rashes lasting 2–6 days every 2 weeks was reported. Moreover, WES demonstrated heterozygous mutation in MVK *c.928G>A and c.1129G>A*.^18^ In the study by Aygun et al. a 16‐month‐old boy was presented with recurrent fever episodes, maculopapular rash and cervical lymphadenopathy. Similar to our report, he had heterozygote mutation of MVK gene, and he was diagnosed as HIDS.^5^

In addition, in patients with mevalonate kinase deficiency‐hyper IgD syndrome (MKD‐HIDS), the enzymatic activity can vary significantly, whereas patients with mevalonate aciduria (MKD‐MA) consistently exhibit very high concentrations of mevalonic acid in their urine. However, the correlation between the severity of MVK mutations and loss of mevalonate kinase enzyme activity remains unclear.^19^ In a study conducted by Jerold Jeyaratnam et al., the diagnostic value of measuring urinary mevalonic acid in patients suspected of having mevalonate kinase deficiency (MKD) was evaluated; while MKD is highly unlikely in patients with normal mevalonic acid excretion, it cannot be completely ruled out. On the other hand, a positive result of urinary mevalonic acid excretion still requires further confirmation through MVK analysis to establish the diagnosis of MKD. Therefore, the detection of urinary mevalonic acid should not be mandatory before genetic testing. However, in situations where genetic testing is not widely available or affordable, measuring urinary mevalonic acid can be a reasonable approach to select patients for MVK gene analysis or enzyme assay.^20^ Furthermore, in their study, Jeyaratnam et al. reported that measuring urinary mevalonic acid demonstrated a sensitivity of 92%, specificity of 90%, positive predictive value of 71%, and negative predictive value of 98%.^20^ Therefore, the presence of consistently high mevalonate urine levels in our case aligns with previous studies highlighting the utility of this biomarker in diagnosing MKD and distinguishing it from other febrile syndromes and these findings underscore the importance of measuring mevalonate urine levels as a diagnostic tool in suspected cases of MKD, allowing for timely intervention and appropriate management strategies.

Cases with severe skin involvement such as those we report here are rare and may lead to misdiagnosis of HIDS as another inflammatory or infectious disorder.^21^ Considering the rarity of HIDS, increasing awareness among healthcare professionals about its clinical presentation, diagnostic criteria, and management is crucial. This will enable early recognition, appropriate intervention, and improved outcomes for patients affected by this syndrome.

Treatment options for Hyper‐IgD syndrome with periodic fever have been a subject of ongoing research and exploration. Although there is currently no known curative therapy for this fever syndrome, various treatment approaches have been investigated to manage symptoms and improve the quality of life for affected individuals.^5^ One of the commonly used treatment strategies for HIDS involves the use of corticosteroids, such as methylprednisolone or prednisolone, to suppress inflammation and control fever episodes. These medications have shown effectiveness in reducing the frequency and severity of fever episodes in many patients. However, long‐term use of corticosteroids may be associated with potential side effects, necessitating careful consideration of their use and close monitoring of patients.^22^ In recent years, the use of biologic agents targeting interleukin‐1 (IL‐1) has emerged as a promising therapeutic approach for HIDS. Anakinra, an IL‐1 receptor antagonist, has demonstrated favorable outcomes in reducing disease activity and improving symptoms in HIDS patients.^5^, ^22^ Studies have reported significant improvements in fever control and reduction in systemic inflammation with the use of Anakinra.^22^ Other IL‐1 inhibitors, such as canakinumab and rilonacept, have also shown efficacy in managing HIDS symptoms.^23^ The choice of treatment may depend on various factors, including the severity of symptoms, individual patient characteristics, and response to therapy.^22^ The decision to initiate biologic therapy should be made on a case‐by‐case basis, considering the potential benefits and risks associated with these medications.^5^, ^22^ It is important to note that while biologic agents targeting IL‐1 have shown promise in managing HIDS symptoms, further research is needed to evaluate their long‐term efficacy, safety, and optimal dosing strategies. Additionally, the cost and accessibility of these medications may pose challenges in certain healthcare settings.^22^, ^23^ Therefore, the treatment of HIDS remains a challenge, with symptomatic management being the primary approach. Corticosteroids have been widely used to control fever episodes, while biologic agents targeting IL‐1, such as Anakinra, have shown promising results in improving symptoms and reducing inflammation. Further research and clinical trials are warranted to establish standardized treatment guidelines and explore novel therapeutic options for individuals with HIDS. A recent study by Yıldız et al. investigated the association between MVK gene polymorphisms and ankylosing spondylitis, another autoinflammatory disorder. While the study did not find a statistically significant difference in MVK gene polymorphisms between patients with ankylosing spondylitis and healthy controls, it highlights the ongoing exploration of the role of MVK in various inflammatory conditions.^24^

## 4 CONCLUSION

The variant in MVK (*C.298G>A*) is a mutation that can lead primary immunodeficiency in patients. Autoinflammatory syndromes always pose diagnostic and therapeutic challenges for therapists. A clinical description of the variety of periodic fever syndromes is useful in the evaluation and management of these patients. Our report highlights the broad clinical phenotype of MVK, and emphasizes the need to consider early genetic screening for young children presenting with attacks of fever associated with skin lesions. Effective management of HIDS involves a multidisciplinary approach in which rheumatologists, immunologists, and geneticists collaborate to provide comprehensive care. Moreover, genetic counseling should be provided to affected individuals and their families to discuss the inheritance pattern and the potential risk of recurrence in future pregnancies.

## AUTHOR CONTRIBUTIONS

**Pooneh Tabibi:** Conceptualization; data curation; writing – original draft; writing – review and editing. **Reza Shiari:** Project administration; supervision; writing – review and editing. **Shabnam Hajiani Ghotb Abadi:** Conceptualization; supervision.

## FUNDING INFORMATION

No funding was received for this research.

## CONFLICT OF INTEREST STATEMENT

The authors declare no conflict of interest regarding the publication of this case report.

## CONSENT

Written informed consent was obtained from the patient to publish this report in accordance with the journal's patient consent policy.

## Data Availability

All data and materials used in this research are available upon request. Researchers interested in accessing the data and materials may contact shiareza@yahoo.com.
